# Piecewise modeling for longitudinal psychological research: a tutorial using R

**DOI:** 10.3389/fpsyg.2026.1949552

**Published:** 2026-10-22

**Authors:** Shuncheng He, Wooyeol Lee

**Affiliations:** Department of Psychology, Chungbuk National University, Cheongju, Chungbuk, Republic of Korea

**Keywords:** latent class growth model, latent transition analysis, longitudinal methods, MAPS, multilevel model, piecewise modeling

## Abstract

Longitudinal psychological research often centers on key developmental transitions, where patterns of change may differ across stages. Piecewise modeling is well suited to capturing these stage-specific changes. This tutorial introduces three piecewise methods in longitudinal psychological research: piecewise multilevel modeling (MLM), piecewise latent class growth modeling (LCGM), and latent transition analysis (LTA). Drawing on a six-wave longitudinal study of national identity development, we show how each method can be implemented in R and highlight the types of research questions each is designed to address, from estimating average developmental trajectories to identifying heterogeneous trajectory patterns and examining transitions between latent developmental states.

## 1 Introduction

Psychological development frequently exhibits non-linear shifts surrounding major transition points, such as educational transitions, treatment phases, or life events (Baltes, 1987; Benner, 2011; Forman-Hoffman et al., 2024). Such stage-dependent shifts are evident across domains, including self-esteem, academic adjustment, identity formation, and clinical outcomes (Eccles et al., 1993; Howard et al., 1993; Huang et al., 2022; Stulz et al., 2007). Piecewise modeling captures this complexity by specifying separate growth parameters for distinct stages defined by theoretical or empirical turning points (Cudeck and Klebe, 2002; Kohli et al., 2018). This tutorial introduces three piecewise modeling approaches, each addressing a distinct research question.

A key consideration in piecewise modeling is whether to specify the changepoint *a priori* or estimate it from the data. A changepoint may be treated as known when theory or study design can specify its location in advance, such as the timing of an educational transition or the onset of an intervention. In this setting, the primary research question concerns whether and how the rate of change differs across predefined phases. In contrast, when a transition is hypothesized, but its location cannot be specified *a priori*, the changepoint can be treated as an unknown parameter and estimated from the data (Kohli and Harring, 2013; Ning and Luo, 2017). This distinction is important because misspecifying a fixed changepoint can bias estimates of growth parameters, while conventional model-fit indices may not reliably detect such misspecification (Ning and Luo, 2017). When the substantive research question concerns detecting whether and where a change occurs, data-driven changepoint detection provides an appropriate alternative (D'Angelo, 2025).

Growth modeling provides a key framework for describing longitudinal change in psychology (Jebb and Tay, 2017). Multilevel modeling (MLM; Singer and Willett, 2003) and latent growth curve modeling (LGCM; Preacher et al., 2008, pp. 185–198) are two primary approaches for estimating population-average trajectories while accounting for individual variation. Specifically, individual differences follow continuous distributions around population means, operationalized as random effects in MLM and latent growth factors in LGCM, respectively. Although MLM and LGCM can yield equivalent results for many conventional growth models, they differ in model parameterization and estimation (Curran, 2003). These differences matter more for intrinsically nonlinear models, such as piecewise models with unknown changepoints, which may require additional reparameterization within the LGCM framework. When theoretical or empirical transitions are anticipated, these frameworks extend to piecewise growth models to capture stage-specific rates of change. Piecewise MLM, for instance, parameterizes distinct average growth rates across predefined developmental phases.

However, not all longitudinal research focuses on population-average trajectories. When a population is expected to comprise multiple latent subgroups rather than a single homogeneous population, person-centered approaches offer a useful alternative. Two widely used methods are piecewise latent class growth modeling (piecewise LCGM; Nagin, 2005) and latent transition analysis (LTA; Collins and Lanza, 2009). Piecewise LCGM identifies latent subgroups that follow qualitatively distinct developmental trajectories. In contrast to piecewise MLM, which models continuous individual differences around a single average trajectory, piecewise LCGM assumes that individuals belong to a finite number of discrete trajectory classes, each with its own stage-specific pattern of change.

Latent transition analysis similarly adopts a person-centered perspective, but it defines a discrete latent status based on cross-sectional response patterns at each measurement occasion, and subsequently estimates transition probabilities between these latent statuses to describe how individuals transition across developmental stages.

While piecewise MLM, piecewise LCGM, and LTA are each well-established, methodological literature often treats them in isolation, focusing primarily on statistical assumptions, model specification, and estimation procedures. Consequently, few studies explicitly compare their conceptual distinctions or the research questions each addresses, offering applied researchers limited guidance when aligning model selection with substantive hypotheses.

Although piecewise LCGM and LTA are widely implemented in proprietary software like Mplus, tutorials demonstrating equivalent workflows in open-source environments remain scarce. The present tutorial focuses specifically on piecewise models with changepoints prespecified based on theory or study design, rather than on the estimation of unknown changepoints from the data. To address this gap, this tutorial demonstrates how to implement piecewise MLM, piecewise LCGM, and LTA in R using longitudinal data centered on a meaningful developmental transition. Beyond technical implementation, we clarify the research questions each method addresses, delineate contexts where each approach is most appropriate, and show how researchers can transition across these frameworks as their research questions evolve. Complete R code and an empirical example are provided to facilitate transparent and reproducible research.

## 2 Example datasets

This study used data from the Multicultural Adolescents Panel Study (MAPS), a nationally representative longitudinal survey conducted annually by the Korean National Youth Policy Institute from 2019 to 2025. The original sample included 2,271 adolescents. To illustrate the proposed modeling approaches, we analyzed six waves of national identity data spanning participants’ middle school (2019–2021) and high school (2022–2024) years. National identity was assessed using four items rated on a 4-point Likert scale: “When someone praises Korea, I feel as if I am being praised,” “I am interested in what people from other countries think about Korea,” “Korea’s success is my success,” and “When someone speaks negatively about Korea, I feel offended as if they were criticizing me.” The transition from middle school to high school was specified as a known changepoint because the six measurement occasions naturally comprised two predefined educational stages: three waves during middle school and three waves during high school. Thus, the study design and the substantive educational transition defined the changepoint rather than estimating it from the national identity data. The descriptive trajectory shown in Figure 1 further supported the relevance of this specification, with national identity generally increasing during middle school and declining following the transition to high school.

**Figure 1:**
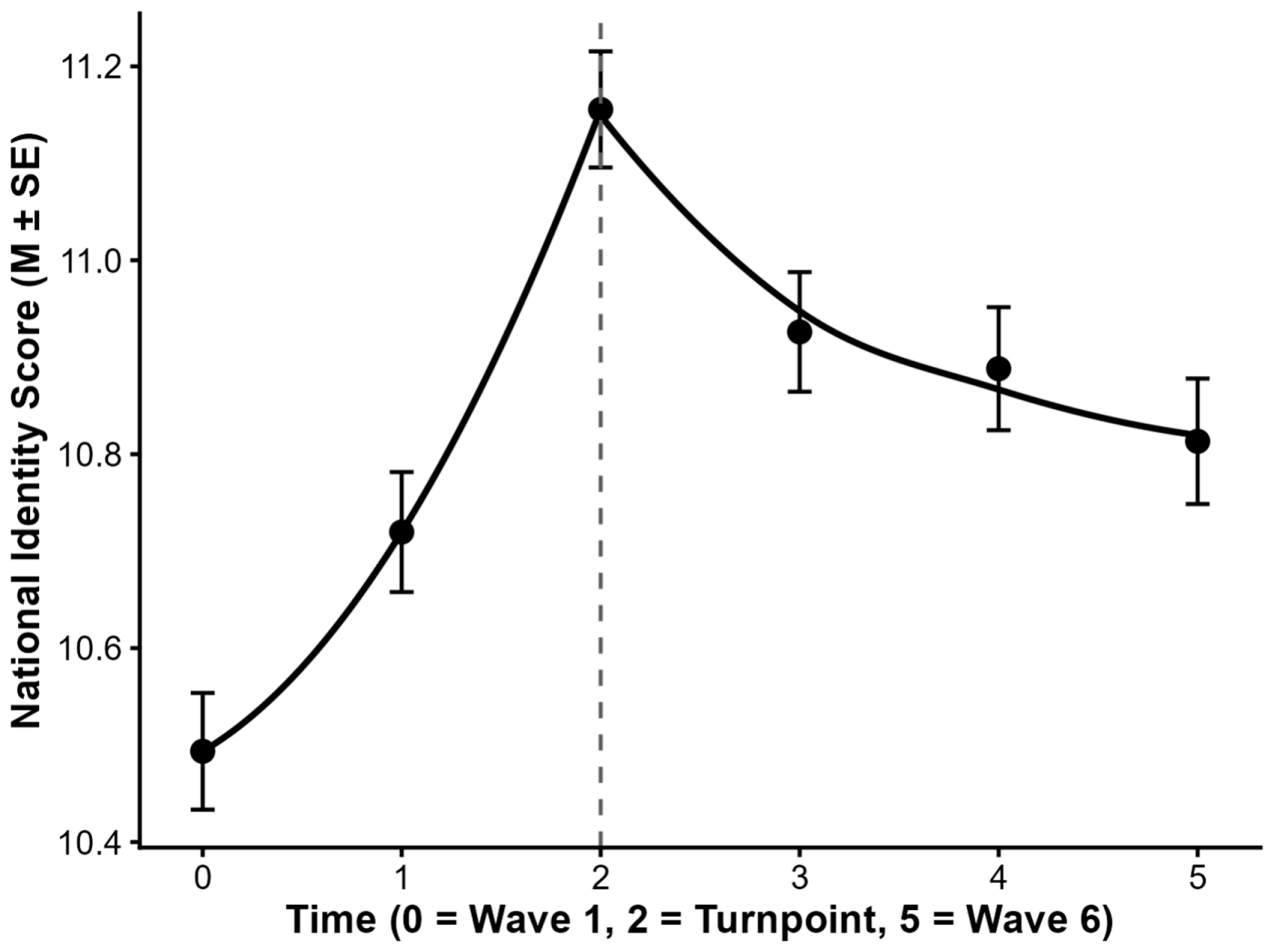
National identity change trend.

## 3 Piecewise multilevel growth model

Although MLM and LGCM differ in their statistical formulations, they generally produce highly comparable results when applied to longitudinal data (Jackson, 2010). Therefore, this study uses a piecewise extended model of the MLM as an example to estimate the rate of change at different development stages.

When two developmental stages are specified, within-person level can be represented as

$$Y_{\mathrm{it}}=\beta_{0i}+\beta_{1i}\mathrm{Tim}e_{1,\mathrm{it}}+\beta_{2i}\mathrm{Tim}e_{2,\mathrm{it}}+e_{\mathrm{it}},$$ (1)

where $Y_{\mathrm{it}}$ is the observed outcome for individual *i* at time *t*, $\beta_{0i}$ is the individual’s initial status (intercept), $\beta_{1i}$ is the slope of change in the first stage [i.e., before the changepoint (middle-school stage)], $\beta_{2i}$ is the slope of change in the two stage[i.e., after the changepoint (high-school stage)], and $e_{\mathrm{it}}$ is the error term, assumed to $e_{\mathrm{it}}\sim N(0,\sigma^{2})$ .

At the between-person level, individual growth parameters are modeled as

$$\begin{array}{l} \beta_{0i}=\gamma_{00}+u_{0i},\\ \beta_{1i}=\gamma_{10}+u_{1i},\\ \beta_{2i}=\gamma_{20}+u_{2i}, \end{array}$$ (2)

where $\gamma_{00}$ , $\gamma_{10}$ , $\gamma_{20}$ denote the average intercept, the average growth rate during the first developmental stage (wave 0–2), and the average growth rate during the second developmental stage (wave 3–5), respectively. The random effects $u_{0i}$ , $u_{1i}$ , and $u_{2i}$ represent individual deviations from the average intercept and two growth rates and are assumed to follow a multivariate normal distribution with a mean vector of zero and the following variance–covariance matrix:

$$(\begin{array}{c} u_{0i}\\ u_{1i}\\ u_{2i} \end{array})∼\mathrm{MVN}((\begin{array}{c} 0\\ 0\\ 0 \end{array}),(\begin{array}{ccc} \tau_{00} & \tau_{01} & \tau_{02}\\ \tau_{10} & \tau_{11} & \tau_{12}\\ \tau_{20} & \tau_{21} & \tau_{22} \end{array})).$$ (3)

The piecewise MLM described above includes both random intercepts and random slopes. If individual variation is assumed to exist only in initial status rather than in rates of change, the model simplifies to a random-intercept model by omitting the random slope terms ( $u_{1i}$ , $u_{2i}$ ). In this constrained specification, $u_{0i}\sim N(0,\tau_{00}\;)$ , and the stage-specific slopes are estimated as fixed effects (i.e., $\beta_{1i}=\gamma_{10}$ , $\beta_{2i}=\gamma_{20}$ ). Alternatively, if development is conceptualized as a single continuous trajectory without distinct developmental transitions, researchers can replace the piecewise specification with a standard linear growth model that fits a single slope across all measurement wave (wave 0–5).

### 3.1 R demonstration

Piecewise MLM were estimated using the *lme4* (Bates et al., 2015) and *lmerTest* (Kuznetsova et al., 2017) packages, while the corresponding piecewise LGCM was estimated using the *lavaan* package (Rosseel, 2012). The R code and data used for these analyses are publicly available on OSF (https://osf.io/r597h/).

**Step 1. Piecewise time coding**


> library(dplyr)
> data_piecewise <− data % > % # long format data
mutate(phase = ifelse(time <= 2, “Phase 1,” “Phase 2”),
time1 = pmin(time, 2), time2 = pmax(0, time - 2))
> head(data_piecewise)
# A tibble: 6 × 7 #time 1 = middle-school stage
PID wave national time phase time1 time2 #time 2 = high-
school stage
1 1832 1 16 0 Phase 1 (0–2) 0 0
2 1832 2  9 1 Phase 1 (0–2) 1 0
3 1832 3 10 2 Phase 1 (0–2) 2 0
4 1832 4 12 3 Phase 2 (2–5) 2 1
5 1832 5 12 4 Phase 2 (2–5) 2 2
6 1832 6 11 5 Phase 2 (2–5) 2 3


The dataset (*data*) must be arranged in long format to fit MLM in R. To capture developmental changes across educational stages, time was modeled using two piecewise variables. The middle school period (Time 1) was coded as 0, 1, 2, 2, 2, and 2 across the six waves, whereas the high school period (Time 2) was coded as 0, 0, 0, 1, 2, and 3. The corresponding coefficients represented growth rates during each stage.

**Step 2. Model fitting**

To evaluate the importance of the piecewise specification, four models were compared. Model 1 included only a random intercept, whereas Model 2 additionally estimated random intercept and random slopes for the middle school and high school phases. Models 3 and 4 ignored the developmental transition by treating the entire study period as a single growth process. Model 3 included only a random intercept, whereas Model 4 also estimated a random slope.


> library(lme4)
> library(lmerTest)
> Model 1 < − lmer(national ~ time1 + time2 + (1| PID),
data = data_piecewise)
> Model 2 < − lmer(national~ time1 + time2 + (1 + time1 +
 time2| PID), data = data_piecewise)
> Model 3 < − lmer(national~ time+ (1|PID),
data = data_piecewise)
> Model 4 < − lmer(national ~ time +(1 + time|PID),
data = data_piecewise)


**Step 3. Model comparison**

All comparisons were conducted using the *anova*() function in R (Bates et al., 2015).


> anova(Model1, Model2, Model3, Model4)
Data: data_long
Models:
Model3: national ~ time + (1 | PID)
Model1: national ~ time1 + time2 + (1 | PID)
Model4: national ~ time + (1 + time | PID)
Model2: national ~ time1 + time2 + (1 + time1 + time2 | PID)
npar AIC BIC logLik −2*log(L) Chisq Df Pr(>Chisq)
Model3 4 55607 55637–27800 55599
Model1 5 55536 55573–27763 55526 73.653 1 < 2.2e-16 ***
Model4 6 55264 55308–27626 55252 274.110 1 < 2.2e-16 ***
Model2 10 55078 55152–27529 55058 193.625 4 < 2.2e-16 ***
---
Signif. codes: 0 ‘***’ 0.001 ‘**’ 0.01 ‘*’ 0.05 ‘.’ 0.1 ‘’ 1


### 3.2 Results and interpretation

Model 2 (piecewise random intercept and random slopes model) yielded the best fit based on Akaike information criterion (AIC; Akaike, 1974) and Bayesian information criterion (BIC; Schwarz, 1978)^1^ indices, supporting distinct developmental trajectories and individual variation across both educational stages.

Results from Model 2 (Table 1) indicated that the average initial level of national identity was 10.47 (*SE* = 0.06, *p* < 0.001). National identity increased significantly during middle school ( $\gamma_{10}$ = 0.31, *SE* = 0.04, *p* < 0.001), but declined during high school ( $\gamma_{20}$ = − 0.10, *SE* = 0.02, *p* < 0.001). Overall, these findings suggest that national identity follows a non-linear developmental trajectory, characterized by growth during middle school and a gradual decline during high school.

**Table 1:** MLM estimation results (Model 2).

Parameter	Estimate	*SE*	*t*	*p*
Fixed effect				
Intercept	10.465	0.058	181.333	<0.001
Time 1	0.307	0.035	8.783	<0.001
Time 2	−0.103	0.023	−4.589	<0.001
Random effect				
Intercept ( $\tau_{00})$	4.286			
Time 1 ( $\tau_{11})$	0.925			
Time 2 ( $\tau_{22})$	0.221			
Corr ( $u_{0j},u_{1j})$	−0.54			
Corr ( $u_{0j},u_{2j})$	−0.34			
Corr ( $u_{1j},u_{2j})$	0.09			
Residual ( σ )	3.898			

Corr = correlation between two random effects; SE = standard error.

The variance of random effects was found for the intercept ( $\tau_{00}$ = 4.29), the middle school slope ( $\tau_{11}$ = 0.93), and the high school slope ( $\tau_{22}\;$ = 0.22), indicating substantial individual heterogeneity in initial status and stage-specific rates of change. Higher initial national identity was associated with slower increases in middle school (*Corr* ( $u_{0j},u_{1j})=$ −0.54) and attenuated declines in high school (*Corr* ( $u_{0j},u_{2j})$ = − 0.34). The correlation between the two slope factors was weak (*Corr* ( $u_{1j},u_{2j})$ = 0.09), suggesting that change during middle school was largely independent of change during high school.

## 4 Piecewise latent class growth model

An important assumption of MLM is that there is a population trend, and that there are individual differences regarding this trend. This assumption may be inappropriate when a population contains qualitatively distinct developmental patterns. For example, if one subgroup shows increasing development while another shows decreasing development, the average trajectory may suggest little or no overall change even though both subgroups exhibit substantial change in opposite directions. Developmental theory likewise suggests that individual differences may reflect qualitatively distinct pathways rather than merely continuous variation in growth rates (Bergman and Magnusson, 1997).

This distinction is often described in terms of variable-centered and person-centered approaches (Muthén and Muthén, 2000). Variable-centered approaches, including regression, multilevel models, and structural equation models, focus on relations among variables and assume that a single population model adequately characterizes all individuals. Person-centered approaches, such as latent profile analysis (LPA) and LCGM, instead seek to identify relatively homogeneous subgroups whose members exhibit similar response patterns, resulting in greater within-class than between-class similarity.

Within this person-centered framework, LCGM extends conventional growth modeling by representing population heterogeneity as a set of discrete latent trajectory classes rather than continuously distributed random effects (Andruff et al., 2009; Jung and Wickrama, 2008). Rather than requiring researchers to specify the number or shape of developmental trajectories in advance, LCGM estimates and compares models with different numbers of latent classes using multiple fit indices to identify the solution that best captures the observed data (Andruff et al., 2009; Nagin, 1999). This feature makes LCGM particularly well suited for exploratory research seeking to identify unobserved developmental heterogeneity within a population (van der Nest et al., 2020).

To understand LCGM, it is first necessary to review the basic framework of LPA. In LPA, observed variables are assumed to follow a normal distribution within each latent class. Specifically, the observed score of individuals (*i*) on variable (*j*) in latent class (*k*) can be expressed as

$$y_{\mathrm{ij}}=\mu_{j}^{\left(k\right)}+\epsilon_{\mathrm{it},}$$

where $\mu_{j}^{\left(k\right)}$ is the class-specific mean of variable (*j*) for latent class (*k*), and $\epsilon_{\mathrm{it}}$ is an individual-specific deviation from that class mean. In LPA, the class-specific mean $\mu_{j}^{\left(k\right)}$ is freely estimated for each observed variable, allowing latent classes to differ in their response profiles across variables. The residual term is assumed to be normally distributed with mean zero and class-specific variance:

$$\epsilon_{\mathrm{ij}}\sim N(0,\sigma_{j}^{2(k)}).$$

In piecewise LCGM, the expected value of the outcome variable is modeled as a time-dependent function of time (*t*). Assuming a linear growth trajectory, the class-specific mean for latent class (*k*) is expressed as

$$\mu_{t}^{\left(k\right)}=\eta_{0}^{\left(k\right)}+\eta_{1}^{\left(k\right)}\mathrm{tim}e_{1,\mathrm{it}}+\eta_{2}^{\left(k\right)}\mathrm{tim}e_{2,\mathrm{it}},$$

where $\eta_{0}^{\left(k\right)}$ is the class-specific intercept, and $\eta_{1}^{\left(k\right)}$ is the class-specific growth rate during the first developmental stage (wave 0–2), and $\eta_{2}^{\left(k\right)}$ is the class-specific growth rate during the second developmental stage (wave 3–5). Incorporating these piecewise growth parameters into the latent profile framework yields the piecewise LCGM formulation:

$$y_{\mathrm{it}}=\eta_{0}^{\left(k\right)}+\eta_{1}^{\left(k\right)}\mathrm{tim}e_{\mathrm{it}}+\eta_{2}^{\left(k\right)}\mathrm{tim}e_{2,\mathrm{it}}+\epsilon_{\mathrm{it}},$$

where $y_{\mathrm{it}}$ is the observed outcome for individual (*i*) at time (*t*), and $\epsilon_{\mathrm{it}}$ is a residual term assumed to follow a normal distribution, $\epsilon_{\mathrm{it}}\sim N(0,\sigma_{t}^{2(k)})$ .

Thus, individuals within the same class share a common developmental trajectory defined by class-specific intercepts and stage-specific slopes. Different classes can therefore exhibit distinct growth patterns across stages, while individual observations vary around these class trajectories due to residual error.

### 4.1 R demonstration

MLM and LCGM can be applied to the same longitudinal dataset because they are designed to model individual developmental trajectories over time. However, they differ fundamentally in their assumptions about between-person heterogeneity.

**Step 1. Fit the baseline one-class model**

To evaluate whether trajectories differed qualitatively across individuals, we first specified a baseline one-class model using the same longitudinal dataset as in the piecewise MLM. This baseline model assumes a single homogeneous population, serving as a benchmark for multi-class comparisons.

The model was estimated using the *hlme()* function from the *lcmm* package (Proust-Lima et al., 2017). Fixed effects were specified for the two piecewise slopes (*time1 and time2*), and a random intercept was included to account for individual differences in initial status.


> lcgm1 < − hlme(fixed = national ~ time1 + time2,
>   random = ~ 1, # random intercept
>  subject = ‘PID’,
>  ng = 1,
>  data = data_ piecewise)


The fixed-effects component was specified as *national ~ time1 + time2*, where *time1* and *time2* estimate the average developmental rates before and after the theoretical transition point, respectively. To establish a baseline developmental trajectory, the one-class model included only a random intercept (*random = ~1*), allowing individual differences in initial levels while assuming a common developmental pattern. Repeated observations were nested within participants (*subject = “PID”*), and the number of latent classes was fixed at one (*ng = 1*). This model served as the baseline for comparing models with additional latent classes and provided starting values for subsequent analyses.

**Step 2. Fit models with increasing numbers of latent classes.**

Following the single-class baseline, models with two through four latent classes were fitted to determine whether multiple distinct trajectories provided a superior fit to the population data. Increasing the number of classes allowed each latent subgroup to estimate its own stage-specific growth parameters.


> lcgm2 < − gridsearch(
>  hlme(fixed = national_iden ~ time1 + time2,
>  mixture = ~ time1 + time2,
>  random = ~ 1,
>  subject = ‘PID’,
>  ng = 2, *## class3, class 4.*>  data = data_piecewise),
>  rep = 30, maxiter = 10, minit = lcgm1)


The *ng* argument specifies the number of latent classes to be estimated. To identify the optimal number of classes, models with increasing numbers of latent classes (e.g., 2, 3, and 4) were estimated and compared. Each model represents a competing hypothesis regarding the underlying heterogeneity of developmental trajectories. The *mixture = ~ time1 + time2* specification allows the fixed effects of *time1* and *time2* to vary across latent classes, enabling each class to have distinct developmental slopes during the middle school and high school periods rather than assuming a common developmental trajectory for all individuals. To minimize convergence to local maxima, all models were estimated using the *gridsearch()* procedure with 30 random sets of starting values and 10 preliminary iterations, and the best-performing solution was retained for final estimation.

**Step 3. Model comparison**

To determine the optimal model, piecewise latent class growth models with one to four classes were evaluated against multiple complementary criteria, including information criteria (e.g., BIC), classification quality, class proportions, and theoretical alignment. Although the four-class solution yielded the lowest BIC, it produced a small class representing only 3.2% of the sample. In contrast, the three-class solution achieved a superior trade-off between statistical fit and substantive interpretability and was thus selected as the final model.


> summarytable(lcgm1, lcgm2, lcgm3, lcgm4)
G loglik npm BIC %class1%class2%class3%class4
lcgm1 1 –27762.90 555564.45100.0000
lcgm2 2 –27591.77 955253.09 16.424483 83.575517
lcgm3 3 –27487.63 1355075.73 84.456187 7.089388 8.454425
lcgm4 4 –27438.84 1755009.06 9.467195 3.170410 7.881990
79.48041


### 4.2 Results and interpretation

As shown in Figure 2 and Table 2, three distinct trajectories of national identity were identified. Class 1 accounted for 8.45% of the sample and displayed a marked increase in national identity. Beginning with the lowest initial level ( η03 = 6.84, *p* < 0.001), adolescents in this class showed substantial gains during middle school ( η13 = 2.43, *p* < 0.001). Growth then plateaued after the transition to high school, with no further significant change detected.

**Figure 2:**
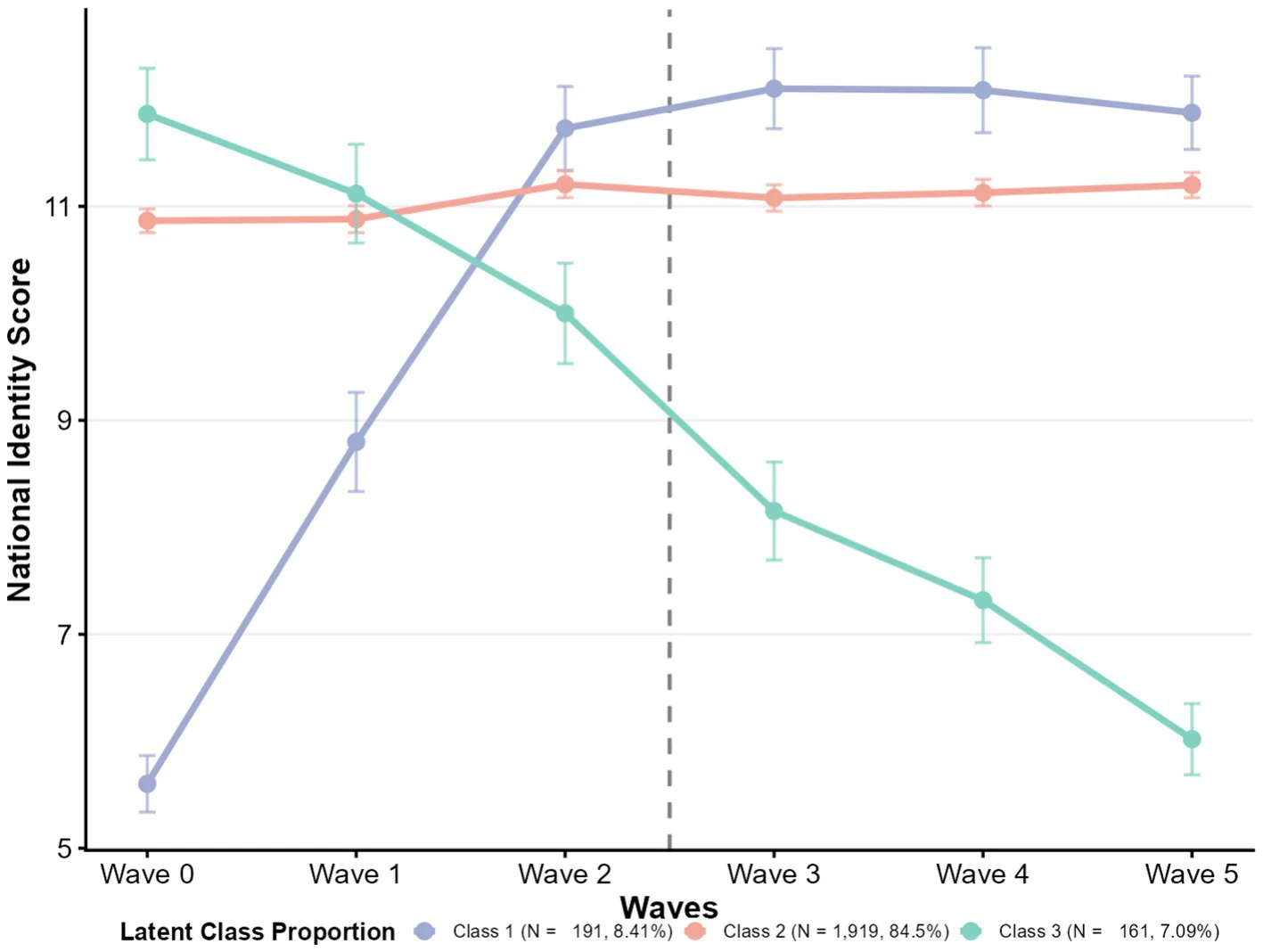
Piecewise LCGA trajectories across middle and high school stages.

**Table 2:** LCGM estimation results (class 3).

Parameter	Class 1, Coef(*p*)	Class 2, Coef(p)	Class 3, Coef(p)
Intercept ( $\eta_{0})$	6.835 (<0.001)	10.844 (<0.001)	11.806 (<0.001)
Time 1 ( $\eta_{1})$	2.433 (<0.001)	0.147(0.004)	−0.918 (<0.001)
Time 2 ( $\eta_{2}$ )	0.079 (0.353)	−0.000 (0.980)	−1.062 (<0.001)
Mixed proportion (%)	8.454	84.456	7.089

Class 2 was the largest group, representing 84.46% of the sample. National identity remained relatively stable over time. Although scores increased modestly during middle school ( η12 = 0.15, *p* = 0.004), no significant change was observed after students entered high school, resulting in an overall stable pattern across the study period.

Class 3, comprising 7.09% of the sample, was characterized by a continuous decline. Adolescents in this class began with the highest level of national identity ( η01 = 11.81, *p* < 0.001), followed by significant decreases during both middle school ( η11 = − 0.92, *p* < 0.001) and high school ( η21 = − 1.06, *p* < 0.001). The rate of decline increased slightly after the transition to high school.

## 5 Latent transition analysis

Latent transition analysis adopts a person-centered perspective by identifying cross-sectional latent states at each measurement wave and estimating the probabilities of transitioning between these states over time. Unlike piecewise trajectory models, LTA does not directly model a continuous developmental trajectory or estimate stage-specific rates of change. Rather, the two approaches address distinct but complementary aspects of longitudinal development: piecewise trajectory models characterize patterns of continuous change across developmental stages, whereas LTA characterizes changes in individuals’ latent-state membership across discrete measurement occasions. Therefore, establishing evidence for a piecewise developmental trajectory is not a prerequisite for conducting LTA. Researchers may conduct LTA independently when their research questions focus specifically on identifying latent states and examining transitions in state membership over time.

Latent transition analysis consists of a measurement model and a structural model. The measurement model is equivalent to an LPA at each measurement wave and identifies latent states based on individuals’ response patterns. At the baseline measurement wave, the observed indicator vector $y_{1i}$ is assumed to arise from a finite mixture of *K* latent classes:

$$P(y_{1i})=\sum_{k=1}^{K}p(c_{1i}=k)p(y_{1i}|c_{1i}=k),$$

Where $P(y_{1i})$ is the marginal distribution of the observed indicator vector for individual *i* at the baseline measurement wave; $p(c_{1i}=k)$ is the probability of membership in latent class *k*, and $p(y_{1i}|c_{1i}=k)$ represents the class-specific conditional distribution of the observed indicators. This formulation assumes that population heterogeneity can be captured by a finite number of unobserved latent classes, each characterized by a distinct response profile. Under the local independence assumption, associations among the observed indicators are assumed to be fully explained by latent-state membership.

To examine whether transition processes differed across developmental periods, two piecewise time variables were included as predictors in the multinomial logistic transition model. The first variable (time 1) captured linear change during waves 0–2 and remained constant thereafter, whereas the second variable (time2) captured linear change during waves 3–5. For a latent transition model consisted of three latent states (*K* = 3), the transition probabilities were modeled using multinomial logistic regression. With the third latent state specified as the reference category, the transition model was formulated as follows:

$$\begin{array}{l} P(C_{\mathrm{it}}=s|C_{i,t-1}=r)\\ =\frac{\exp(\alpha_{r,s}+\beta_{1,r,s}\text{time}1_{t}+\beta_{2,r,s}\text{time}2_{t})}{\sum_{m=1}^{3}\exp(\alpha_{r,m}+\beta_{1,r,m}\text{time}1_{t}+\beta_{2,r,m}\text{time}2_{t})}, \end{array}$$

where $P(C_{i,t}=s|C_{i,t-1}=r)$ is the probability of transitioning from latent state (*r*) at time (*t-1*)to latent state (*s*) at time (*t*); $S_{t-1}$ and $S_{t}$ are the latent states at the previous and current time points, respectively; $\alpha_{r,s}$ is the intercept for the transition from state (*r*) to state (*s*); $\beta_{1,r,s}$ and $\beta_{2,r,s}$ represent the effects of the two piecewise time variables (Time 1 and Time 2) on the transition probability; $\sum_{m=1}^{3}\exp(\alpha_{r,m}+\beta_{1,r,m}\text{time}1_{t}+\beta_{2,r,m}\text{time}2_{t})$ is the normalization term, which sums the exponentiated linear predictors across all three possible destination states to ensure that the transition probabilities from a given origin state sum to one. The coefficients associated with the reference state were fixed to zero for model identification.

This specification extends the standard time-homogeneous transition model by incorporating two piecewise time predictors, allowing transition probabilities to vary across developmental stages. As a result, the model can evaluate whether the likelihood of moving between latent states differs systematically across the middle and high school developmental periods.

### 5.1 R demonstration

Unlike MLM and LCGM, which model repeated outcome scores, LTA estimates latent states using observed indicators (i.e., item-level responses). Therefore, the data should be organized in long format, with each row representing one individual at one measurement wave, as shown below.


head(lta_piecewise)
PID wave national_01 national _02 national _03 national
_04 wave1 wave2
1 1832 1 4 4 4 4 0 0
2 1832 2 2 3 2 2 1 0
3 1832 3 2 3 3 2 2 0
4 1832 4 3 3 3 3 2 1
5 1832 5 3 3 3 3 2 2
6 1832 6 3 3 2 3 2 3


**Step 1. Determine the number of latent states using LPA**

Following Ryoo et al. (2018), fit separate latent class/profile models at each measurement occasion to identify a plausible range of candidate status solutions rather than determine a single number of statuses for the subsequent LTA. These preliminary solutions can be evaluated using statistical fit indices (e.g., AIC and BIC), classification quality, and substantive interpretability. The resulting candidate solutions can then be compared within the longitudinal LTA framework, with the final number of latent statuses selected based on both model-fit information and conceptual interpretability (Lanza et al., 2010).

Before conducting the LTA, LPAs were performed separately at each of the six-measurement wave to identify an appropriate latent profile structure. The LPAs were conducted using the tidyLPA package (Rosenberg et al., 2018).


> library(tidyLPA)
> lpa_wave1 < − lta_piecewise_wave 1% > % estimate
profiles(n_profiles = 1:4, models = 1)
> lpa_wave2 < − lta_piecewise_wave2% > % estimate
profiles(n_profiles = 1:4, models = 1)
……
>lpa_wave6 < − lta_piecewise_wave6% > % estimate
profiles(n_profiles = 1:4, models = 1)


After conducting LPAs separately at each of the six-measurement wave, the optimal number of latent profiles was identified. These profiles are conceptually different from the latent classes identified by piecewise LCGM. Piecewise LCGM groups individuals according to differences in their developmental trajectories over time, whereas LPA groups individuals according to similarities in their responses to the observed indicators at a given measurement wave.

**Step 2. Fit models**

After identifying candidate profile solutions based on the preliminary LPA results, LTA models with different numbers of latent states were fitted and compared using AIC and BIC. The final state solution was selected based on model fit and substantive interpretability.


> library(depmixS4)
> ntimes <− table(df_lta$PID) # Define participant-specific
 sequence lengths
> forms <− lapply(items, function(x) as.formula(paste(x, “~ 
1”)))# form define
> lta_model_3states < − depmix(
 response = forms,
 data = lta_piecewise,
 ntimes = as.numeric(ntimes),
nstates = 3, ## state 2–4
transition = ~ wave1 + wave2,
family = rep(list(multinomial(“identity”)), 4)


The LTA was estimated using the *depmixS4* package (Visser and Speekenbrink, 2010). The *response* argument specifies *forms,* a list of intercept-only response formulas (~ 1) for the four observed indicators used to define the latent states in the LTA model. Setting *nstates = 3* fitted a three-state model, in which individuals were assigned to one of three latent developmental states at each measurement wave. Transition probabilities were modeled as a function of the two piecewise time variables (*wave1 + wave2*), allowing transition patterns to differ between the two developmental stages rather than remain constant across the study period. The *family* argument, *rep(list(multinomial(“identity”)), 4)*, specified multinomial distributions for all four observed indicators, treating them as categorical variables.

### 5.2 Results and interpretation

As shown in Figure 3, the LPA results supported the three-profile solution across all six waves. Although the four-state LTA showed slightly better fit than the three-state solution (Table 3), the additional state primarily reflected a further differentiation of the lower level of national identity rather than a qualitatively distinct pattern. In contrast, the three-state solution provided a clearer and more parsimonious representation of low, moderate, and high levels of national identity. Considering statistical fit, substantive interpretability, and parsimony together, the three-state model was retained. As presented in Table 4, the three states were characterized by distinct levels of national identity across the four indicators. State 1 (36.9%) exhibited the highest indicator scores and was labeled *low identity*, State 2 (47.1%) exhibited intermediate scores and was labeled *moderate identity*, and State 3 (16.1%) exhibited the lowest scores and was labeled *high identity*.

**Figure 3:**
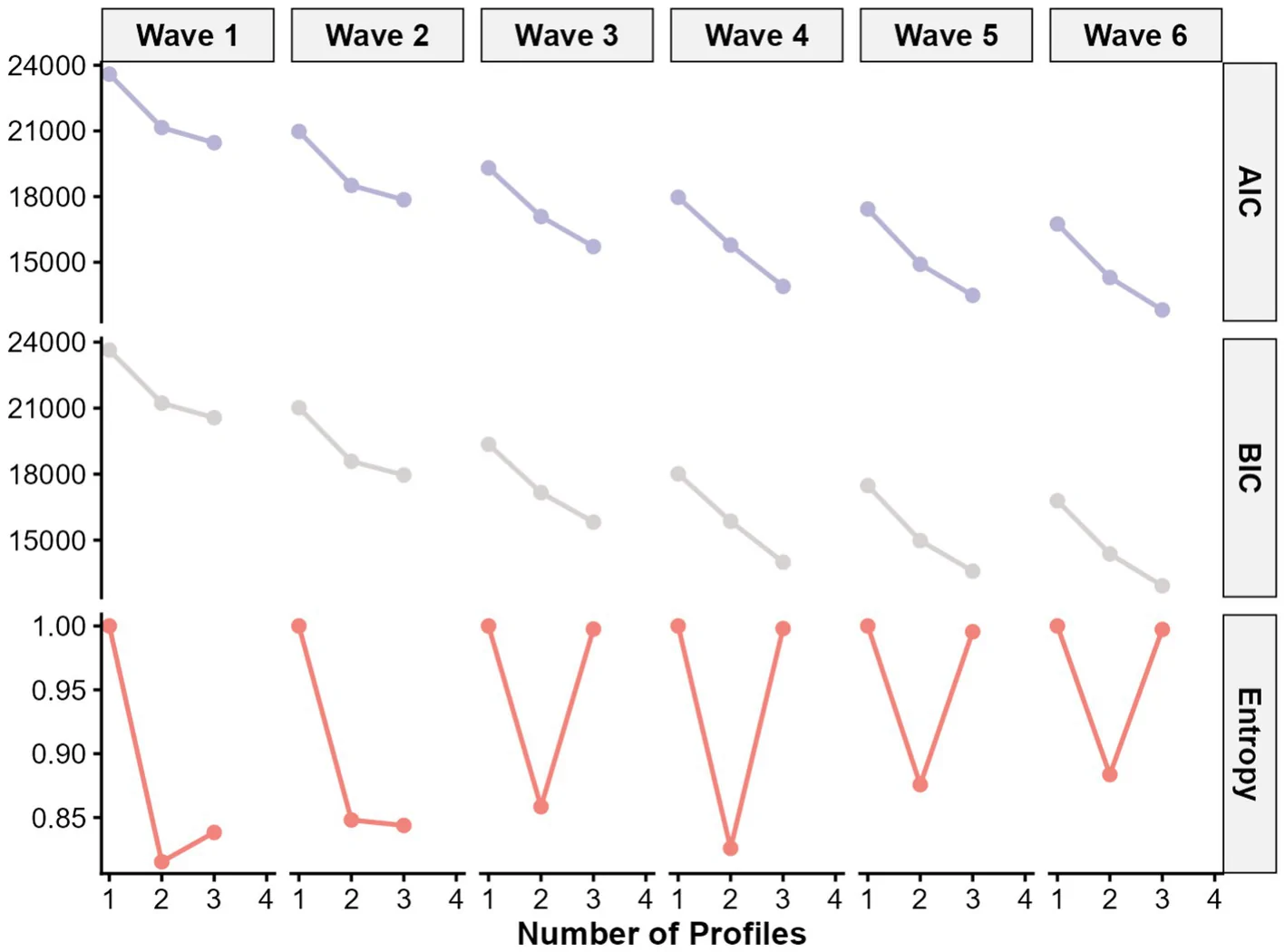
Model fit indices across latent profile solutions.

**Table 3:** Model fit indices across LTA.

Model	LogLik	AIC	BIC
Two-state model	−56575.80	113213.59	113446.70
Three-state model	−51638.78	103389.56	103810.67
Four-state model	−49148.48	98470.97	99125.18

**Table 4:** Three latent states in the LTA.

State	Item 1	Item 2	Item 3	Item 4	Group
State 1(36.9%)	1.913	1.880	1.943	1.883	Low
State 2(47.1%)	2.959	2.841	2.879	2.825	Moderate
State 3(16.1%)	3.788	3.554	3.747	3.638	High

Figure 4 illustrates the estimated transition probabilities for the middle school and high school periods. The moderate identity state exhibited the greatest stability in both periods, with the probability of remaining in this state increasing from 0.72 in middle school to 0.78 in high school. Stability also increased for the high identity state (0.51 to 0.53) and the low identity state (0.58 to 0.67), indicating greater persistence of all three identity states during high school.

**Figure 4:**
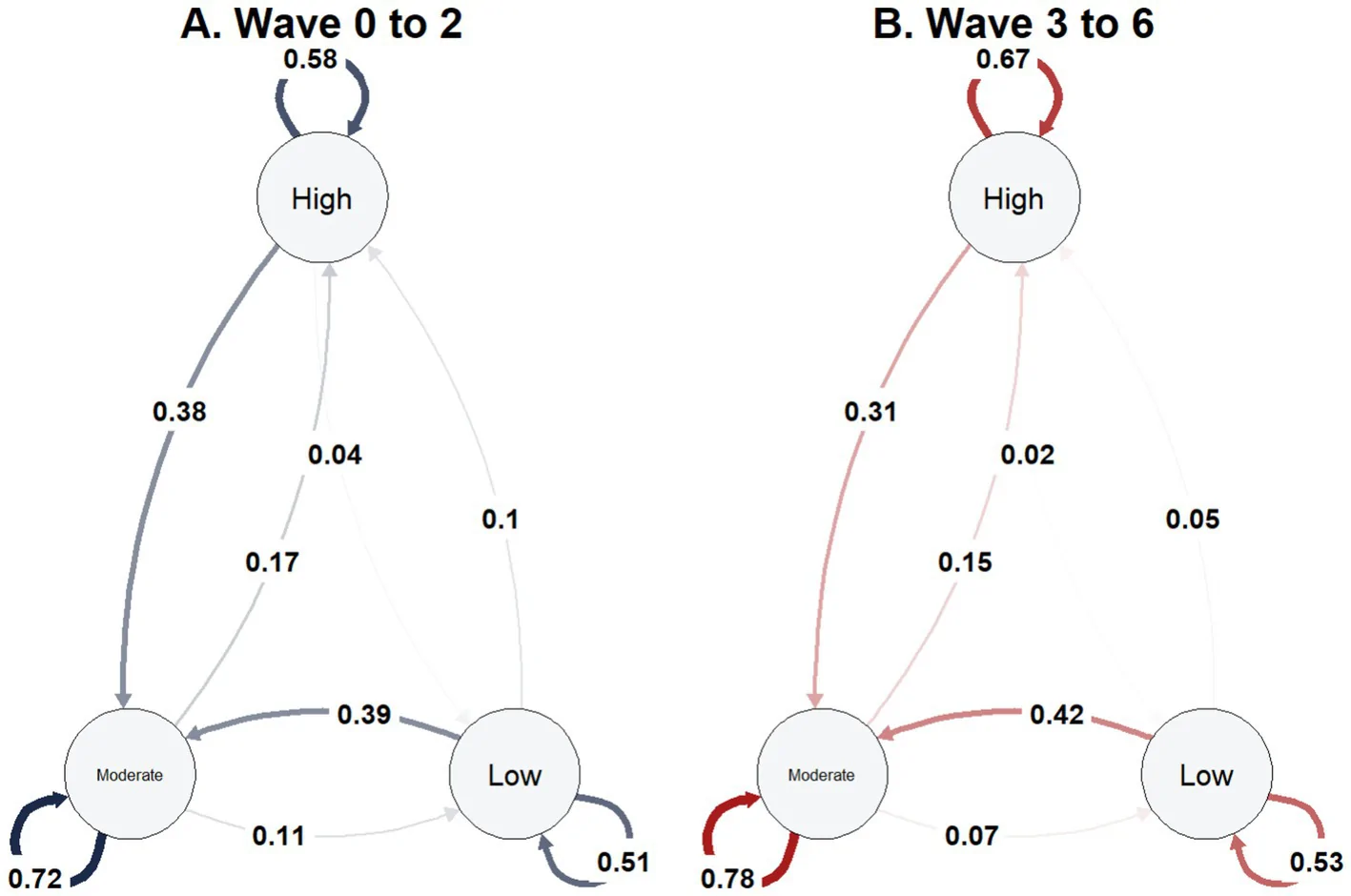
LTA probabilities across stages: **(A)** middle and **(B)** high school.

Transitions aligned predominantly toward the moderate state rather than between extreme states. High-identity adolescents increasingly transitioned to the moderate state across periods (0.39 to 0.42), while low-to-moderate transitions remained consistently frequent (0.38 and 0.31). Direct transitions between high and low identity states were negligible. Overall, the results indicate that adolescents’ national identity became increasingly centered on the moderate identity state. Compared with the high and low identity states, the moderate identity state exhibited the greatest Stability and accounted for the largest share of transitions across both educational stages.

## 6 Discussion

This tutorial demonstrates piecewise MLM, piecewise LCGM, and LTA as complementary frameworks for modeling stage-dependent developmental processes. Using national identity as an empirical illustration, we show how these methods address distinct but complementary aspects of longitudinal change: average stage-specific growth, trajectory heterogeneity, and transition dynamics across latent states. These methods move beyond overall mean trajectories to capture discrete sub-groups and their evolving state transitions over time.

### 6.1 Application issues

The models described in this tutorial are most suitable for longitudinal studies that examine theoretically meaningful transition points, include repeated assessments spanning at least two developmental stages, and address questions about average developmental change, developmental heterogeneity, or transitions between developmental states. In these settings, a single growth model is often insufficient to represent stage-specific developmental processes, and choosing an analytical method is an important consideration.

In practice, researchers should also consider sample size requirements, which increase with model complexity. Simulation studies suggest that LCGM generally requires larger samples as the number of latent classes increases, class separation decreases, and missing data become more prevalent, with recommended sample sizes ranging from approximately 200 for two-class solutions to 1,000 for five-class solutions (Kim, 2012; Tein et al., 2013). For LTA, sample size requirements also depend on the number of measurement wave, transition probabilities, class separation, and class sizes, with previous studies generally recommending sample sizes of 300–1,000 and at least 500 participants for most applications (Finch and Bronk, 2011; Nylund-Gibson and Choi, 2018).

Although MLM, LCGM, and LTA are based on different latent variable assumptions, they provide complementary perspectives on the same developmental process. MLM characterizes developmental change as continuous variation across individuals. LCGM captures developmental heterogeneity by identifying distinct trajectory classes. LTA focuses on changes in latent developmental states over time. Considered together, these models represent continuous latent variables, categorical latent classes, and dynamic latent states, respectively. This allows researchers to study development issues from different perspectives.

### 6.2 Method extension

Compared with dedicated software, the open-source R environment offers considerable flexibility and integration for longitudinal analyses. Researchers can address missing data using multiple imputation (e.g., Multiple Imputation by Chained Equations) and incorporate time-varying or analysis-specific covariates. Beyond the packages demonstrated in this tutorial, several R packages offer alternative implementations of longitudinal modeling frameworks, each with different strengths and limitations. Table 5 summarizes representative R packages for MLM, LGCM, LCGM, and LTA and highlights their main advantages and limitations.

**Table 5:** *R* Packages for different longitudinal modeling frameworks: advantages and limitations.

Research focus	Method	R package	Advantages	Limitations
Continuous trajectory	Piecewise MLM	*lme4* (Bates et al., 2015)	Flexible specification of fixed and random effects; straightforward implementation of piecewise trajectories	Limited specification of within-person residual covariance structures; does not provide fit indices
		*nlme* (Pinheiro et al., 2025)	Allows flexible residual covariance structures and nonlinear mixed-effects models	Model specification can be more complex than in lme4
	Piecewise LGCM	*lavaan* package (Rosseel, 2012)	Provides SEM-based global fit indices; allows growth parameters to be represented as latent factors	Complex piecewise models may require additional parameterization, particularly when the changepoint is unknown
Trajectory heterogeneity	Piecewise LCGM	*lcmm* package (Proust-Lima et al., 2017)	Designed for latent-class mixed models; accommodates class-specific longitudinal trajectories	Requires careful selection of the number of classes and initial values
		*flexmix* for LCGM (Grün and Leisch, 2008)	Provides a flexible framework for finite-mixture regression models	Not specifically designed for longitudinal growth modeling; complex growth structures require additional specification
Status transition	LTA	*mixtools* for LTA (Benaglia et al., 2010)	Provides flexible tools for finite-mixture modeling and latent-class estimation	Does not directly implement conventional LTA; transition structures require additional programming
		*depmixS4* package (Visser and Speekenbrink, 2010)	Supports longitudinal latent-state models and estimation of transition probabilities	Primarily based on the hidden Markov modeling framework

For ordinal or categorical longitudinal outcomes, researchers may consider alternative modeling approaches depending on their research questions. Researchers can implement generalized linear mixed-effects models using R packages such as *lme4* and *glmmTMB*. In contrast, researchers can estimate latent class growth or mixed models with *lcmm* when the focus is on heterogeneous developmental trajectories. Alternatively, LTA, a longitudinal extension that links latent class analyses (LCAs) across multiple time points, can examine transitions between latent states (Nylund-Gibson et al., 2023). Although R packages such as *OpenMx* and *mixtools* can implement *LTA*, they have limitations for more complex specifications. Mplus provides a more flexible framework for specifying and estimating such models (Nylund-Gibson et al., 2023).

Piecewise modeling is not limited to two linear segments separated by a single changepoint and can represent more complex developmental processes. For example, different developmental phases may follow different functional forms, including nonlinear trajectories, and models may incorporate multiple changepoints when more than two phases of change are expected (Harring et al., 2021). Piecewise models can also accommodate more complex longitudinal data structures. For example, piecewise crossed random-effects models can account for dynamic group membership over time while modeling nonlinear growth (Rohloff et al., 2024). Extensions to multivariate settings include bivariate piecewise models, which jointly model two segmented longitudinal processes and their associations over time (Peralta et al., 2022). Other extensions include piecewise growth models that separate state and trait components of change (Heo et al., 2026) and models for examining mediation processes across developmental stages (Liu and Perera, 2023). Mixture mediation models provide another extension by accounting for population heterogeneity in longitudinal mediation processes (Zhang et al., 2026). Together, these extensions illustrate the flexibility of piecewise modeling for addressing nonlinear change, complex data structures, and relationships among multiple developmental processes.

## Data Availability

Data collected from the Multicultural Adolescents Panel Study (MAPS) by the National Youth Policy Institute were used in this study. Data are available at the following: https://www.nypi.re.kr/archive/mps. Further inquiries can be directed to the corresponding author/s.

## Footnotes

1 Lower values of AIC and BIC indicate a better trade-off between model fit and parsimony.
